# Silent complication: chronic diarrhea as a clue to gastrojejunocolic fistula: A case report

**DOI:** 10.1097/MD.0000000000043577

**Published:** 2025-08-01

**Authors:** Mariane Ghantous, Zeinab El Zein, Rached Radwan, Antoine Geagea, Houssam Alam, Antoine Abou Rached

**Affiliations:** a Department of Hepatogastroenterology, Lebanese University, Beirut, Lebanon; b Department of General Surgery, Lebanese University, Beirut, Lebanon.

**Keywords:** Billroth II procedure, chronic diarrhea, gastrojejunocolic fistula

## Abstract

**Rationale:**

Gastrocolic fistulae represent abnormal connections between the stomach and large intestine, often leading to chronic diarrhea and malnutrition. They can arise postoperatively, particularly after complex surgeries such as the Billroth II procedure.

**Patient concerns:**

This case report describes the case of a 71-year-old male, with a history of hypertension and gastrointestinal surgery, who experienced significant weight loss and postprandial diarrhea. Initial investigations, including stool cultures and multiple imaging studies, failed to reveal the fistula.

**Diagnoses:**

A definitive diagnosis of gastrojejunocolic fistulae was made during the third gastroscopy, when the endoscope passed from the stomach into the colon.

**Interventions:**

Surgical intervention involved adhesiolysis and resection of the fistula, followed by reconstruction of gastrointestinal continuity.

**Outcomes:**

Postoperative care included nutritional support, and the patient showed a significant improvement at follow-up.

**Lessons:**

This case highlights the diagnostic challenges associated with gastrojejunocolic fistulae and emphasizes the need for a high index of suspicion in patients with atypical gastrointestinal symptoms after complex surgery. This illustrates that timely surgical intervention can lead to positive outcomes, and underscores the importance of ongoing research and education regarding the management of such complications. Further studies are needed to understand the long-term effects of gastrojejunocolic fistulae and optimize management strategies.

## 1. Introduction

A gastrocolic fistula is an abnormal pathological connection between the epithelialized mucosal layers of the stomach and large intestine. A connection typically forms between the greater curvature of the stomach and distal section of the transverse colon.^[1]^ Such fistulae can lead to chronic diarrhea, malnutrition, and significant morbidity.^[2]^ Gastrocolic fistulae often arise from inflammatory conditions, malignancies, or surgical complications and may present insidiously, complicating timely diagnosis and management.^[3]^ These fistulae are rare but significant complications of gastrointestinal surgery, particularly after Billroth II gastrectomy for peptic ulcer disease.^[2]^

The Billroth II procedure, often performed for peptic ulcer disease and gastric malignancies, can lead to gastrocolic fistula formation, typically due to stomal ulceration caused by incomplete gastric resection, insufficient vagotomy, or a long afferent loop.^[2]^ Recent literature highlights the diagnostic challenges posed by gastrocolic fistulae, as symptoms can mimic those of other gastrointestinal disorders, often leading to treatment delays.^[1,4]^ Chronic diarrhea, as a presenting symptom of gastrocolic fistula, has been documented in various case reports, underscoring the importance of clinical awareness.^[5,6]^

This case report describes a 71-year-old patient who developed a gastrojejunocolic fistula 16 years after antrectomy with Billroth II reconstruction for peptic ulcer. His presentation of chronic diarrhea and weight loss was explored in the context of the current literature to elucidate the mechanisms, diagnosis, and management of this uncommon postoperative complication.

## 2. Case presentation

A 71-year-old male with a history of hypertension presented 16 years after undergoing antrectomy with Billroth II reconstruction, which was later converted to Roux-en-Y owing to surgical complications from intractable peptic ulcer disease. He complained of severe watery diarrhea that had persisted for several months, with periods of complete symptom resolution. The patient reported experiencing severe watery diarrhea immediately after meals for the past 4 months, which significantly disrupted his daily routine. As a result, he planned meals around his activities, avoiding eating before outings, to prevent postprandial diarrhea. He also noted an approximate weight loss of 10 kg over 3 months due to his symptoms. Additionally, the patient reported episodes of fecal belching associated with diarrhea.

**Figure 1.**
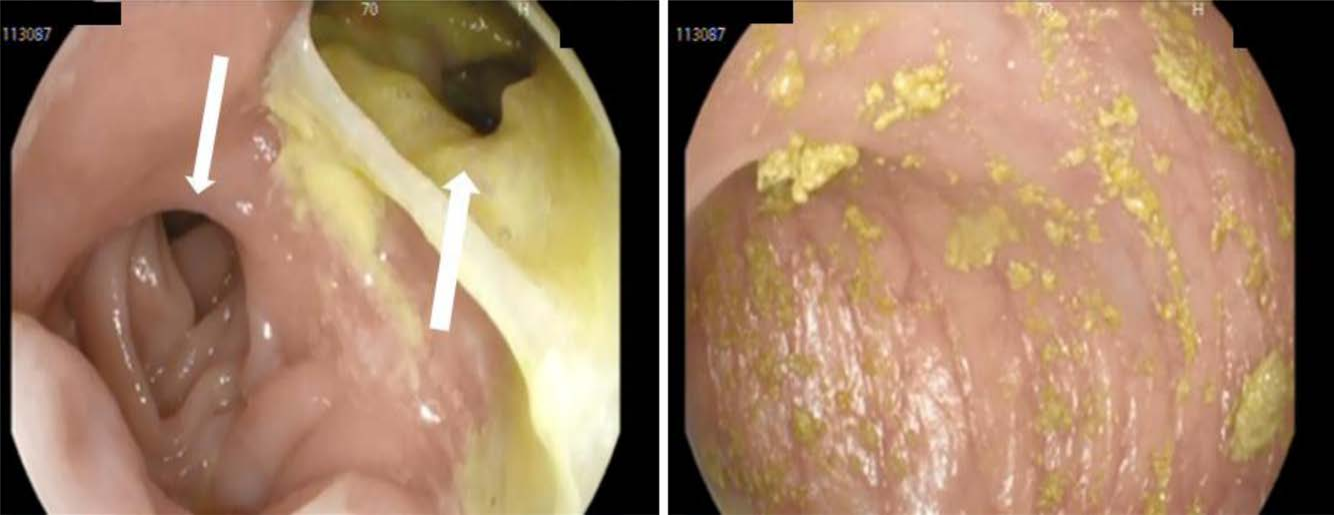
To the left: Gastroscopy revealing the gastrojejunostomy (downward pointing arrow) and gastric-colonic fistula with fluid and fecal material in the stomach (upward-pointing arrow). To the right: Gastroscopy showing a large gastrocolic fistula with easy endoscopic passage into the colon.

**Figure 2.**
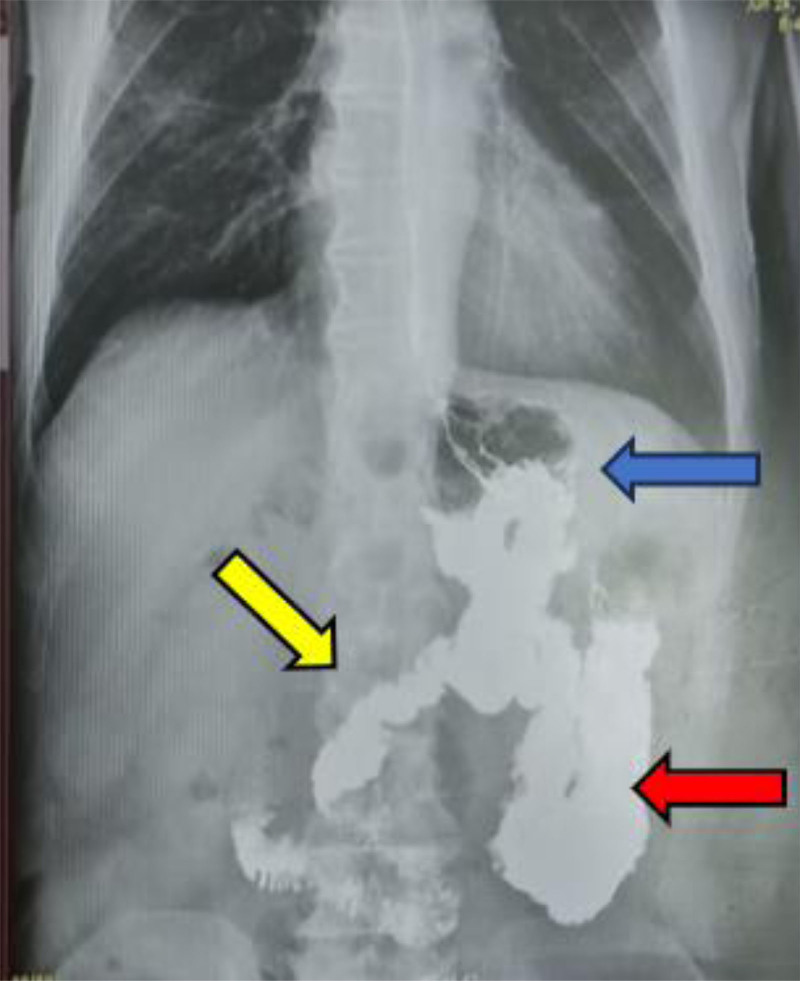
Barium swallow: transit of barium contrast from the stomach (blue arrow) to the jejunum (yellow arrow) and transverse colon (red arrow) simultaneously indicating the GCF. GCF = gastrocolic fistula.

He has initially seen by multiple healthcare practitioners but to no avail. Multiple healthcare practitioners prescribed a course of Rifaximin and Metronidazole. These medications did not alleviate his symptoms. He also took Loperamide to decrease the number of episodes of diarrhea; however, his symptoms persisted. He underwent a vigorous workup that included many clinical testings. Stool analysis and culture studies, done in an outpatient setting, were negative for any infectious processes. A computed tomography (CT) of the abdomen and pelvis with intravenous contrast study, also done in an outpatient setting, was negative for any significant findings and revealed only surgical staples at the site of gastrojejunostomy and jejunojejunostomy. A magnetic resonance imaging (MRI) with gadolinium also did not give conclusive results, and the patient had already undergone 2 gastroscopies and colonoscopies, which did not reveal ulcerations or other findings before presentation. A definitive third attempt at a gastroscopy revealed the passage of the endoscope from the stomach into the colon after sudden visualization of colonic mucosa during gastroscopy (Fig. 1). Hereafter, a barium swallow was ordered which showed the passage of the barium contrast from the stomach and into the jejunum and colon simultaneously (Fig. 2).

The patient was diagnosed with a gastrojejunocolic fistula, explaining his symptomatology. Thus, operative intervention was decided to be the definitive treatment for this patient. A preoperative nutritional assessment revealed no hypoalbuminemia or electrolyte disturbances. However, further blood tests revealed leukocytosis with left shift. The patient received peripheral parenteral nutrition 24 hours before the operation. In addition, bowel preparation using Sodium Picosulfate was administered per os 24 hours before the surgery.

**Figure 3.**
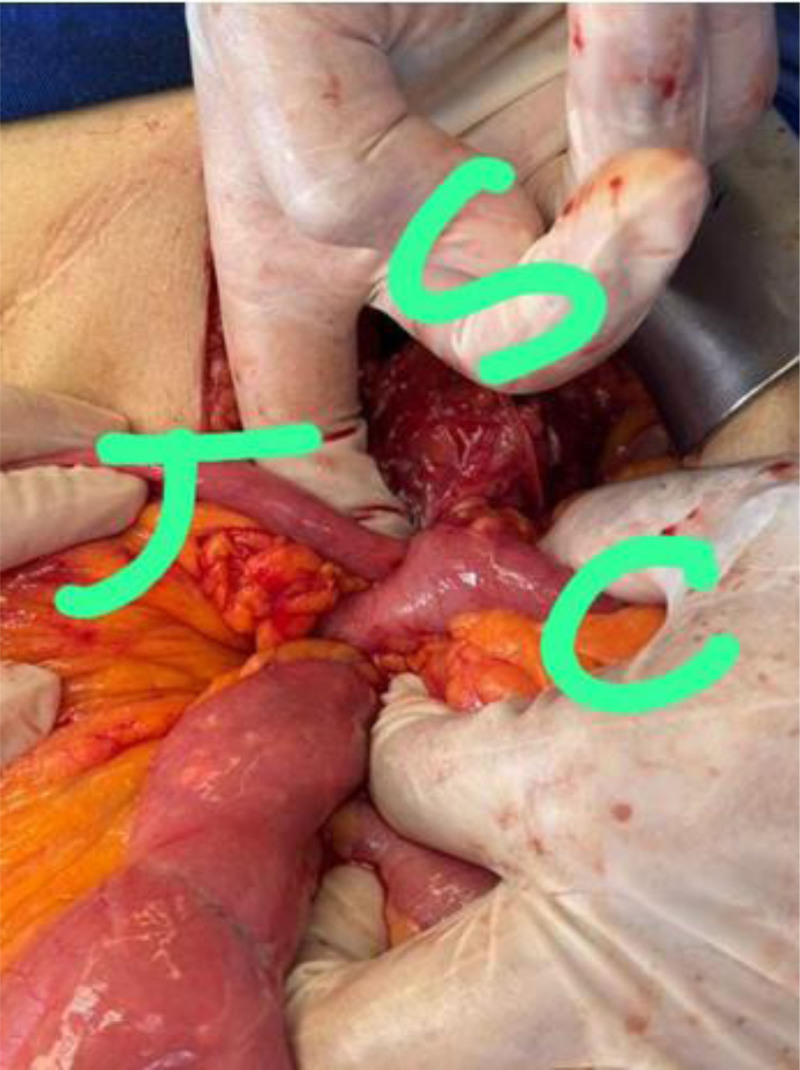
Intraoperative findings: presence of communication between the gastrojejunostomy and transverse colon indicating the GCF. GCF = gastrocolic fistula.

**Figure 4.**
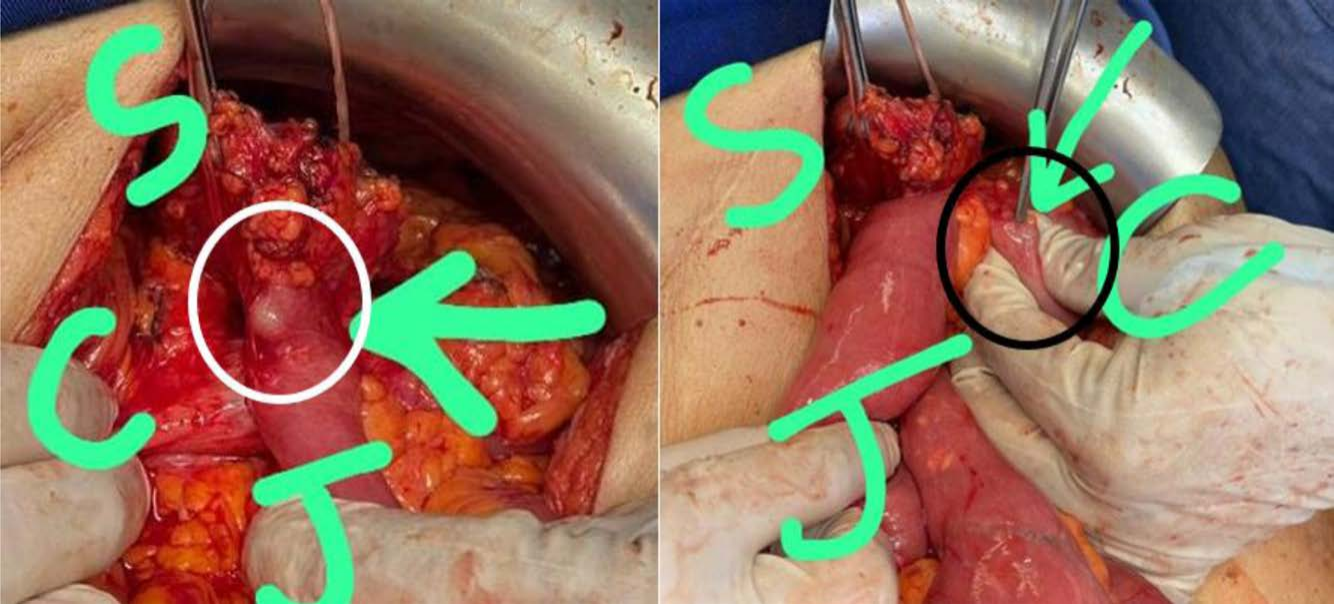
Intraoperative findings: gastrojejunocolic fistula documented by spontaneous passage of nasogastric tube from stomach into jejunum (white circle; left picture) and NGT passage from stomach into the colon (black circle; right picture). C = colon, J = jejunum, S = stomach.

**Figure 5.**
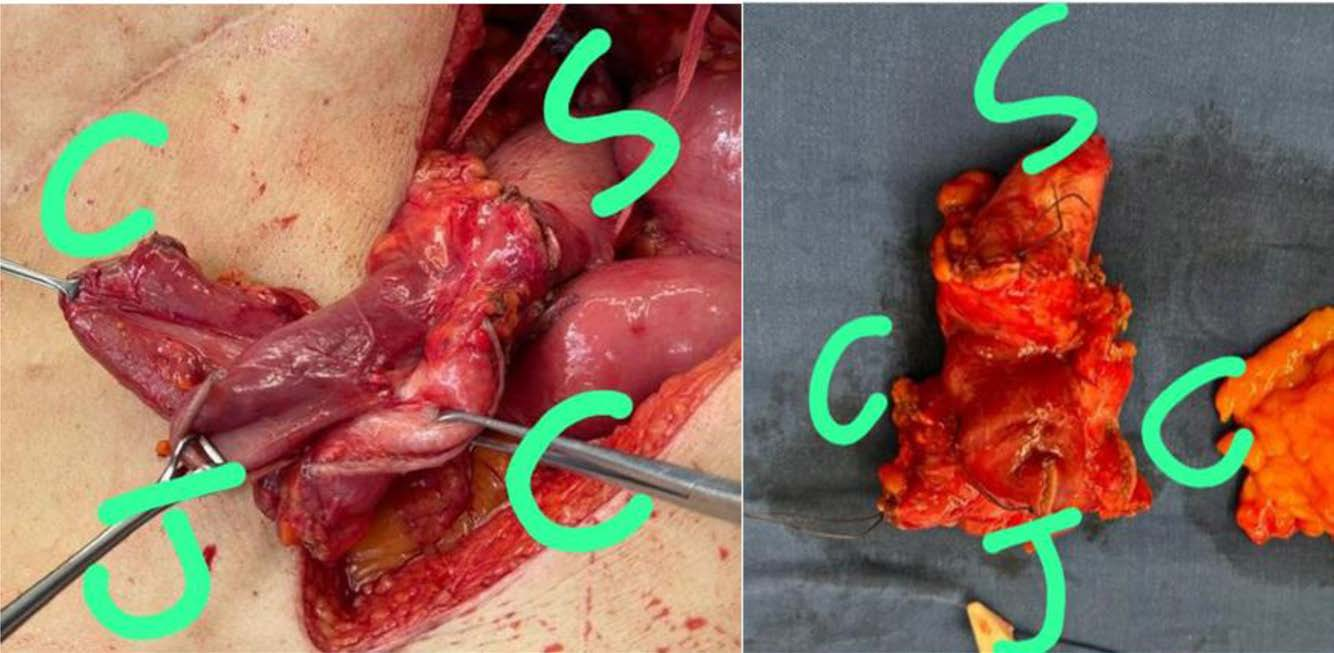
Intraoperative findings: To the left is the complex fistula before en bloc resection is completed. Right: En bloc resection of the gastrojejunostomy fistula with a specimen on the draped table. C = proximal and distal transverse colon ends, J = jejunum, NGT = nasogastric tube, S = stomach.

The patient was placed on the operating table in a supine position with arms extended outwards. The surgical plan was to identify the gastrojejunocolic fistula and resect it with any associated adhered inflamed tissue before reestablishing gastrointestinal tract continuity. A vagotomy was also discussed, given the patient’s complex history of peptic ulcer disease. Under general anesthesia, a midline laparotomy through the previous incision was done. After careful adhesiolysis using Ligasure and electrocautery, communication between the gastrojejunostomy and transverse colon was observed (Figs. 3 and 4). En bloc resection of the fistula complex was performed by applying three 60-mm purple gastrointestinal staplers to the transverse colon proximal and distal to the fistula, and 1 GIA stapler was applied at the stomach cephalad to the original gastrojejunostomy. Reconstruction of the colon was done first by establishing a side-to-side colocolic anastomosis using an 80-mm purple GIA stapler (Fig. 5). The gastrojejunostomy was recreated using a 60-mm black GIA stapler with oversewing of the stapler line using vicryl 3.0. Owing to extensive adhesions and thickening of the stomach tissue, the vagotomy was aborted despite it being discussed as part of the initial surgical plan. The abdomen was then closed in the appropriate manner after a hemovac drain was placed near our anastomoses. The patient was initially transferred to the ICU for a 24-hour period monitoring, which was unremarkable. He was then transferred to a regular floor where he received a broad-spectrum antibiotic with total parenteral nutrition for 3 days until enteral feeding was appropriately adequate. He started on sips of water on day 2 postop with gradual escalation in diet according to tolerance. During his hospital stay, the hemovac did not drain anything significant. It was removed on day 4 postop. On the fifth post operative day the patient was discharged home on a proton pump inhibitor and analgesics with no complications. Pathological examination of the excised specimen revealed only inflammatory changes, with no evidence of malignancy.

Initial 2-week postop follow-up revealed complete healing of surgical incision and staplers used to close the incision at the time of surgery were then removed. In the clinic, a 4-month postoperative follow-up and the patient reported to be doing very well. He has been tolerating regular food requiring no restrictions, and his diarrhea has resolved. He has also been compliant to the proton pump inhibitor, taking esomeprazole 40 mg once daily, and leading a healthy lifestyle with no recurrence of his previous symptoms.

## 3. Discussion

Gastrojejunocolic fistulas are a recognized but rare complication of surgeries that alter the gastrointestinal anatomy, such as Billroth II gastrectomy.^[2]^ Previous studies have demonstrated that these fistulas can arise because of several factors including surgical trauma, chronic inflammation, and ischemia at the surgical site.^[1,4]^ In this case, the patient presented with a 16-year delay between the initial surgery and the onset of symptoms, highlighting the long-term risks associated with complex gastrointestinal surgeries.

The literature shows growing awareness of gastrojejunocolic fistulas as a potential complication of various gastrointestinal surgeries, including gastrojejunostomy and gastric bypass.^[7,8]^ Documented cases indicate that these fistulas can present either early or many years after surgery, as in our case, in which the fistula manifested 16 years after surgery. This underscores the importance of routine monitoring and educating patients regarding the recognition of postoperative symptoms.^[3,6]^

This case emphasizes the need to maintain a high index of suspicion for fistula formation even in patients who are far removed from their original procedure. Chronic postprandial diarrhea, fecal belching, and significant weight loss strongly indicate direct communication between the stomach and colon, which is characteristic of gastrojejunocolic fistulas.^[5,6]^ Although these symptoms are distinctive, they can easily be misinterpreted as more common postoperative gastrointestinal issues, contributing to delays in diagnosis. The rapid onset of diarrhea after eating observed in this patient suggests a rapid transit of gastric contents into the colon, a hallmark of gastrointestinal fistulas.^[2,9]^

The diagnostic challenges in this case are significant. Despite severe postprandial diarrhea and fecal belching, multiple diagnostic tests failed to identify the fistula early. Stool analysis and culture studies did not reveal an infectious cause, and imaging studies such as CT and MRIs were inconclusive. The complexity of postoperative changes, such as adhesions and altered anatomy, likely obscured the fistula or made interpretation of imaging difficult. In gastrojejunocolic fistulas, traditional imaging techniques may have limited sensitivity owing to the presence of surgical staples, scar tissue, or fistula size.^[1]^ In this case, the CT did not provide clear evidence due to extensive postoperative changes, and MRI did not offer additional insights. This mirrors the findings in the literature, where initial imaging often fails to detect significant abnormalities in cases of gastrojejunocolic fistulas.^[3,10]^

Although endoscopy is a critical tool for diagnosing gastrointestinal fistulas, it can yield false-negative results. In this case, the patient underwent gastroscopy and colonoscopy, both of which failed to detect a fistula. This suggests that the location or extent of the fistula may not have been accessible or visible during the initial examination. The eventual discovery of the fistula during the third gastroscopy was pivotal in reaching a diagnosis. This finding reinforces the need for repeated and thorough endoscopic evaluations, especially when clinical suspicion remains high despite the negative findings. Once endoscopy suggested the presence of a fistula, a barium swallow test was performed to confirm the diagnosis. The passage of contrast from the stomach into both the jejunum and colon simultaneously provides definitive evidence of gastrojejunocolic fistula. Barium swallow studies are particularly effective in visualizing abnormal transit patterns, making them a crucial diagnostic tool when a fistula is suspected. This case clearly demonstrated abnormal communication and helped guide the preoperative planning by mapping the course of the fistula.

Currently, there are no established guidelines for the treatment of gastrojejunocolic fistulas. However, previous case reports have demonstrated that definitive management is typically achieved through surgical intervention, specifically en bloc resection of the fistula, as illustrated in our case.

Several key lessons can be drawn from this case for clinicians managing potential fistulas, particularly in postsurgical patients.

1. The persistent pursuit of a diagnosis is essential, even when initial studies are normal. The fact that both the initial gastroscopy and colonoscopy missed the fistula highlights the importance of repeat testing when clinical suspicion remains high.
2. However, imaging studies may not provide early evidence. A high index of suspicion should prompt further diagnostic testing, including contrast studies and repeat endoscopies.
3. Barium studies are invaluable for visualizing and mapping fistulas.
4. Thus, an interdisciplinary approach is required. Early involvement of gastroenterologists, surgeons, and radiologists can lead to a more comprehensive diagnostic process.

This case underscores the importance of considering fistula formation as a differential diagnosis in patients with a history of gastrointestinal surgery who present with atypical symptoms, such as chronic diarrhea. Timely diagnosis and intervention can significantly improve patient outcomes, as demonstrated by the patient’s full recovery and resolution of symptoms at the 4-month follow-up.

## 4. Conclusion

Chronic diarrhea in patients with a history of gastric surgery should raise the suspicion of gastrocolic or gastrojejunocolic fistula formation. Early and accurate diagnosis followed by prompt surgical intervention is critical for symptom resolution and improvement of patient outcomes. Ongoing research is needed to optimize the management of these rare but serious complications.

## Author contributions

**Conceptualization:** Mariane Ghantous.

**Data curation:** Mariane Ghantous, Zeinab El Zein, Houssam Alam, Antoine Abou Rached.

**Formal analysis:** Mariane Ghantous.

**Investigation:** Mariane Ghantous, Rached Radwan.

**Methodology:** Zeinab El Zein, Mariane Ghantous.

**Resources:** Mariane Ghantous.

**Writing – original draft:** Mariane Ghantous, Zeinab El Zein.

**Writing – review & editing:** Mariane Ghantous, Zeinab El Zein, Antoine Abou Rached.

**Supervision:** Antoine Geagea, Houssam Alam, Antoine Abou Rached.

**Validation:** Antoine Abou Rached.
